# Co-treatment of tumor cells with hyaluronan plus doxorubicin affects endothelial cell behavior independently of VEGF expression

**DOI:** 10.18632/oncotarget.26379

**Published:** 2018-11-27

**Authors:** Daiana L. Vitale, Fiorella M. Spinelli, Daiana Del Dago, Antonella Icardi, Gianina Demarchi, Ilaria Caon, Mariana García, Marcela F. Bolontrade, Alberto Passi, Carolina Cristina, Laura Alaniz

**Affiliations:** ^1^ Laboratorio de Microambiente Tumoral-Centro de Investigaciones y Transferencia del Noroeste de la Provincia de Buenos Aires (CIT NOBA, UNNOBA-CONICET), Junín, Buenos Aires, Argentina; ^2^ Laboratorio de Fisiopatología de la Hipófisis-Centro de Investigaciones y Transferencia del Noroeste de la Provincia de Buenos Aires (CIT NOBA, UNNOBA-CONICET), Junín, Buenos Aires, Argentina; ^3^ Laboratorio de Terapia Génica, IIMT–CONICET, Universidad Austral, Derqui-Pilar, Buenos Aires, Argentina; ^4^ Laboratorio de Células Madre-Instituto de Biología y Medicina Experimental (IBYME-CONICET), Buenos Aires, Argentina; ^5^ Dipartimento di Medicina e Chirurgia, Universitá degli Studio dell’Insubria, Varese, Italia

**Keywords:** hyaluronan, cancer, doxorubicin, tumor microenvironment, angiogenesis

## Abstract

Hyaluronan, the main glycosaminoglycan of extracellular matrices, is concentrated in tissues with high cell proliferation and migration rates. In cancer, hyaluronan expression is altered and it becomes fragmented into low-molecular-weight forms, affecting mechanisms associated with cell proliferation, invasion, angiogenesis and multidrug resistance. Here, we analyzed the effect of low-molecular-weight hyaluronan on the response of T lymphoma, osteosarcoma, and mammary adenocarcinoma cell lines to the antineoplastic drug doxorubicin, and whether co-treatment with hyaluronan and doxorubicin modified the behavior of endothelial cells. Our aim was to associate the hyaluronan-doxorubicin response with angiogenic alterations in these tumors. After hyaluronan and doxorubicin co-treatment, hyaluronan altered drug accumulation and modulated the expression of ATP-binding cassette transporters in T-cell lymphoma cells. In contrast, no changes in drug accumulation were observed in cells from solid tumors, indicating that hyaluronan might not affect drug efflux. However, when we evaluated the effect on angiogenic mechanisms, the supernatant from tumor cells treated with doxorubicin exhibited a pro-angiogenic effect on endothelial cells. Hyaluronan-doxorubicin co-treatment increased migration and vessel formation in endothelial cells. This effect was independent of vascular endothelial growth factor but related to fibroblast growth factor-2 expression. Besides, we observed a pro-angiogenic effect on endothelial cells during hyaluronan and doxorubicin co-treatment in the *in vivo* murine model of T-cell lymphoma. Our results demonstrate for the first time that hyaluronan is a potential modulator of doxorubicin response by mechanisms that involve not only drug efflux but also angiogenic processes, providing an adverse tumor stroma during chemotherapy.

## INTRODUCTION

Hyaluronan (HA), a large linear polysaccharide, is the main glycosaminoglycan found in all types of mammalian extracellular matrices (ECM). HA is able to interact with cell surface receptors such as CD44 and RHAMM, activating different cellular signals [1, 2]. It is well known that, in malignant tumors, HA expression is altered in comparison to normal tissues [3]. In fact, during cancer transformation, HA is fragmented into low-molecular-weight (LMW) forms, which have been shown to promote cell proliferation, adhesion and motility, and are considered tumor growth promoters [4]. High expression of CD44, the main HA receptor, is associated with a normal and tumor stem cell-like phenotype [5]. In this sense, tumor stem cells currently exhibit high resistance to chemotherapeutic agents, since they also present an increased expression of different multidrug resistance ATP-binding cassette (ABC) transporters, such as ABCB1 (P-glycoprotein), ABCC1, and ABCG2, which modulate cytotoxic drug efflux [6–8]. Some works have shown that the CD44-HA interaction affects the function of drug transporters by several mechanisms, including the modulation of their gene expression and activity [9]. Opposite to the action of native HA, it has been demonstrated that HA fragments sensitize vincristine-resistant lymphoma cell lines by modulating P-glycoprotein activity and the PI3K/Akt survival pathway [10–12].

HA is also an important factor involved in tumor angiogenesis. This molecule promotes the formation of tumor-associated vasculature by inducing the expression of different angiogenic factors such as vascular endothelial growth factor (VEGF) and the fibroblast growth factor (FGF). These processes activate a continuous angiogenesis and different oncogenic pathways, inhibition of apoptosis and acquisition of a tumor stem cell-like phenotype [13]. Moreover, it has been observed that, during treatment with the antineoplastic drug doxorubicin (DOX), tumor-associated endothelial cells (ECs) acquire resistance to the antiangiogenic drug sunitinib [14].

Regarding the oncogenic pathway, the canonical Wnt/β-catenin and PI3K/Akt signaling pathways have been linked with many types of cancers. It has been documented that Wnt signaling regulates CD44 expression and function, and reciprocally CD44 targets the Wnt pathway [15–17]. Aberrant activation of the PI3K/Akt signaling pathway leads to tumor cell survival, and several studies have demonstrated that the HA-CD44 interaction sustains the activation of phosphorylated Akt (p-Akt) signaling and modulates tumor progression [12]. These pathways result in an interesting potential therapeutic target to inhibit metastasis, drug resistance and recurrence processes [18–21].

Tumor-associated ECs may develop drug resistance through the regulation of different cellular pathways; in this way, they also need to be eliminated by chemotherapy treatment to minimize tumor progression and risk of recurrence [8].

In the present work, we studied the effect of HA on DOX treatment in different tumor cell lines and evaluated the modulation of DOX accumulation as well as the activation of Wnt and PI3K/Akt pathways. We also analyzed the impact of these treatments on ECs behavior. Our data indicate that the presence of LMW HA in tumor stroma could negatively influence the response to chemotherapy treatment, in part by inducing β-catenin expression and p-Akt in tumor and associated stromal cells. Importantly, we observed functional alterations in ECs, which affected the angiogenic response and thus affect the success or failure of the tumor treatment.

## RESULTS

### CD44 expression and HA binding in tumor cells

To study whether EL4 (murine T-cell lymphoma) and K12 (murine osteosarcoma) cell lines were able to respond to HA treatment, we first analyzed CD44 expression and HA binding ability in these tumor cell lines.

**Figure 1:**
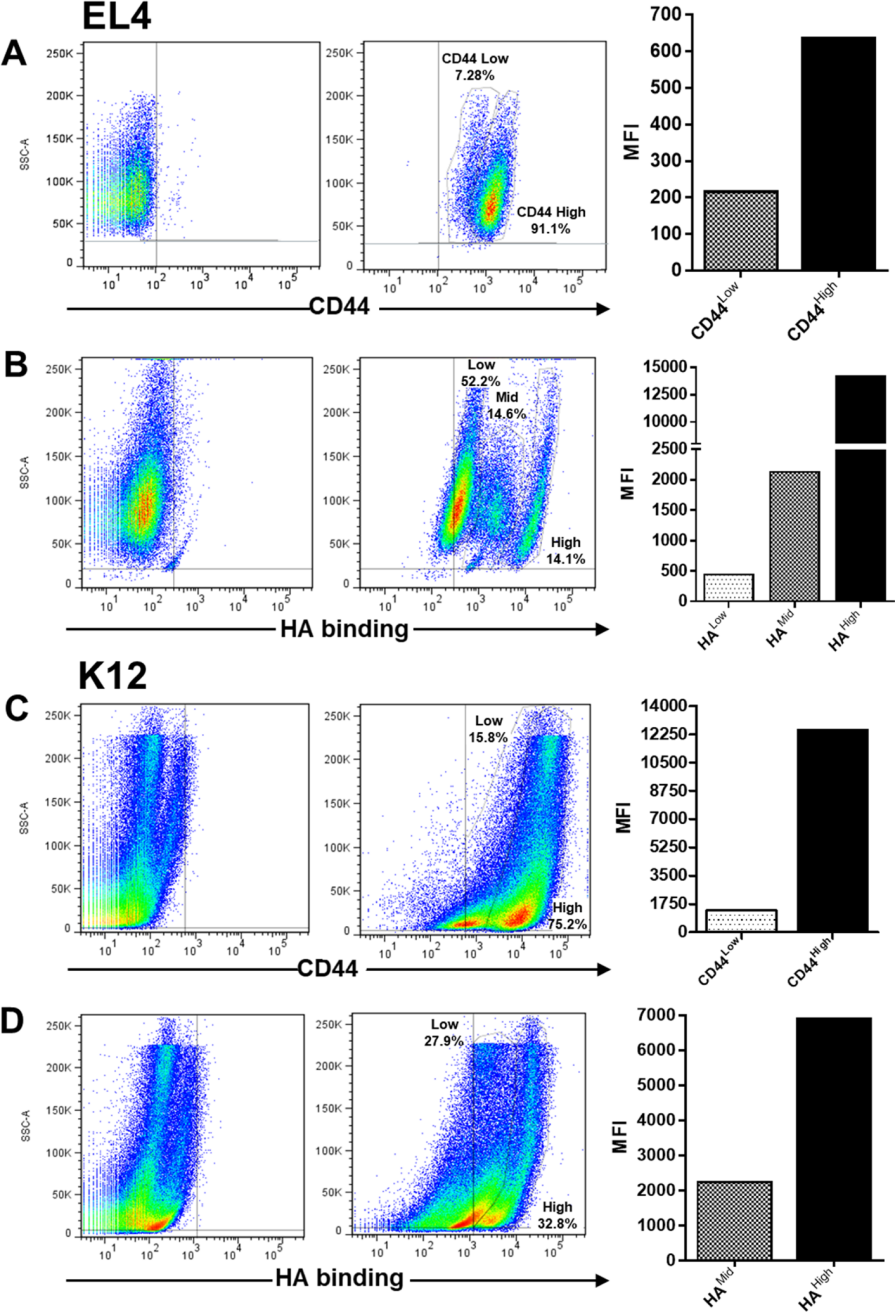
Flow cytometry analysis of CD44 expression and hyaluronan (HA) binding ability in EL4 (**A** and **B**) and K12 (**C** and **D**) cell lines. Graphs show the Mean Fluorescence Intensity (MFI) of cell populations with different CD44 expression level and HA binding ability, representative of three independent experiments.

Regarding CD44 expression, EL4 cells showed two cell populations with different mean fluorescence intensity (MFI) of CD44: one main population with high CD44 expression (MFI: 641) and a small population with lower CD44 expression (MFI: 219) (Figure 1A), whereas regarding HA binding ability, EL4 cells presented three populations which bound HA with different MFI levels (HA^high^: 14300, HA^mid^: 2144, HA^low^: 460) (Figure 1B).

Similarly, the K12 osteosarcoma cell line showed two populations with different CD44 expression levels (CD44^high^: 12500 and CD44^mid^: 1347) (Figure 1C), which bound HA with different MFI levels (HA^mid^ 2144, HA^high^: 6941) (Figure 1D).

As it is known, the MDA-MB-231 cell line is used as a model of invasive breast cancer, expressing high levels of CD44 and binding HA efficiently [22]. Thus, we also studied this cell line and found high CD44 and HA binding levels by flow cytometry (data not shown).

### Effect of LMW HA-DOX co-treatment on drug accumulation, expression of ABC drug transporters and cell death

To analyze the effect of HA as a modulator of drug response in tumor cells, we used DOX because it is used to treat a broad spectrum of solid tumors (breast adenocarcinoma, osteosarcoma, bronchogenic carcinoma, and neuroblastoma) as well as hematologic malignancies (lymphomas and acute leukemia) [23]. Previous works have shown the ability of ECM components, such as HA, to modulate drug resistance [24, 25]. Considering these previous data, we decided to evaluate the potential effect of exogenous HA (mimicking the tumor microenvironment) on DOX accumulation and apoptosis induction in the three tumor cell lines mentioned above.

Different doses of DOX (0.5 µM; 1 µM; and 2.5 µM) in combination with LMW HA (20 µg/ml and 100 µg/ml) were used to evaluate DOX accumulation by flow cytometry analysis. DOX doses were selected considering values below the IC50 for each cell line (Supplementary Figure 1: IC50_EL4_: 2.4 µM; IC50_K12_: 6.8 µM; IC50_MDA-MB-231_: 4.9 µM) to avoid effects of high levels of cell death. The presence of functional drug efflux pumps was confirmed by using the specific ABC transporter inhibitor Cyclosporine A (CsA) in the three cell lines (data not shown).

**Figure 2:**
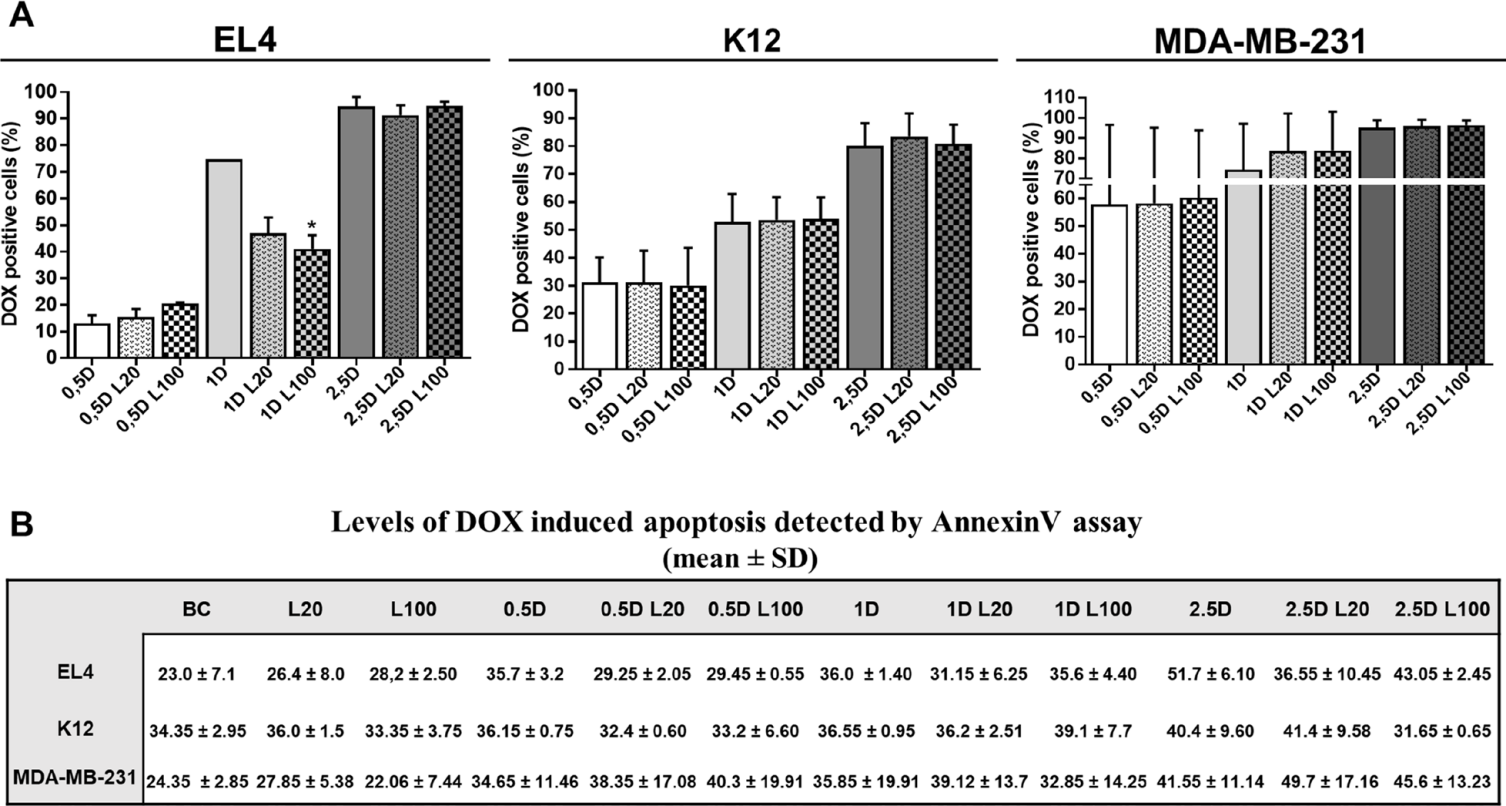
Effect of LMW HA on drug accumulation and cell death Flow cytometry analysis of doxorubicin (DOX) accumulation (**A**) and apoptosis (**B**) evaluated in EL4, K12 and MDA-MB-231 cell lines. Cells were incubated with 0.5, 1 and 2.5 µM of DOX alone (0.5 D, 1 D and 2.5 D) or plus 20 or 100 µg/ml of LMW HA (L20 and L100 respectively). To measure apoptosis levels, cells were labeled with AnnexinV-FITC and analyzed by flow cytometry. Values are expressed as arithmetic means ± standard deviation (SD) evaluated in three independent experiments. ^*^p < 0.05, ^**^p < 0.01 vs. untreated cells.

A significant reduction of DOX intracellular levels was observed in EL4 cells only when 1 μM DOX was combined with 100 µg/ml LMW HA, whereas no changes in DOX accumulation were observed in K12 or MDA-MB-231 cells (Figure 2A). In agreement, no significant differences in DOX-induced apoptosis were found after DOX and HA co-treatment in all cell lines (Figure 2B).

**Figure 3:**
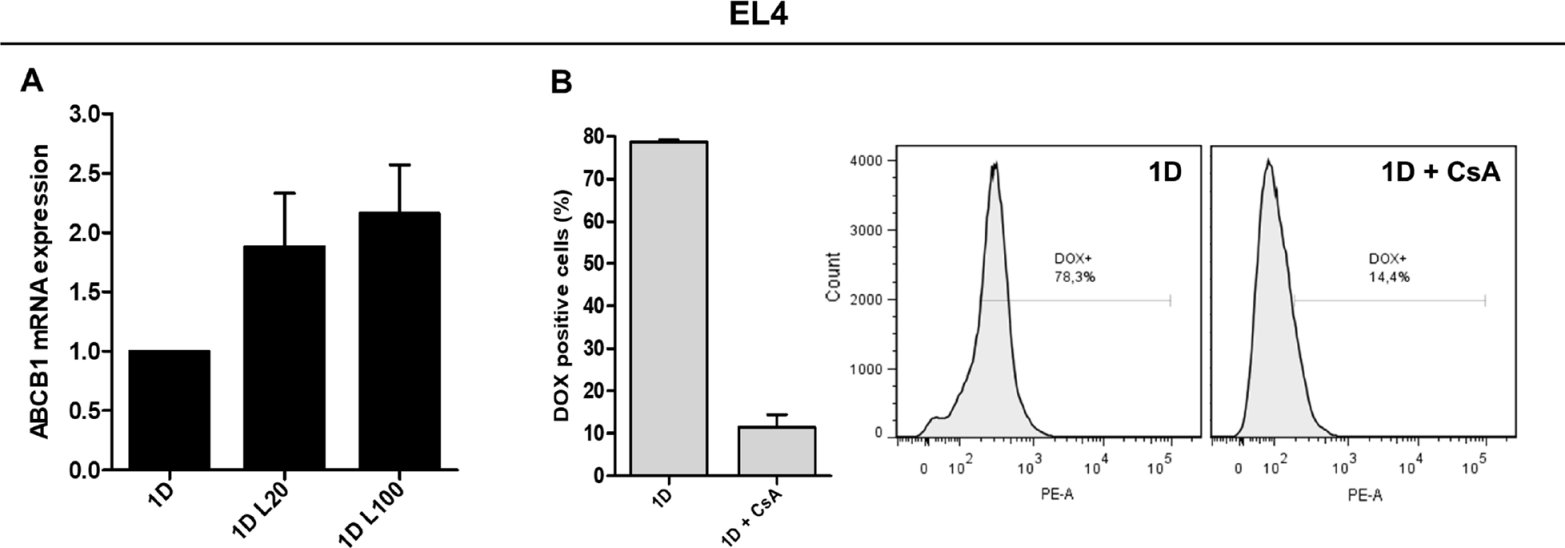
Expression and function of drug efflux pumps in response to LMW HA and DOX co-treatment ABCB1 mRNA quantification by RT-qPCR in EL4 cells after DOX and HA co-treatment. GAPDH mRNA expression was used as reference gene (**A**). The function of drug efflux pumps in EL4 cells was evaluated studying DOX accumulation in the presence of 100 µM of the blocking agent Cyclosporine A (CsA) (**B**). Results are expressed as means ± SD obtained in three independent experiments. ^*^p < 0.05, ^**^p < 0.01 vs. untreated cells.

Since we observed differences in DOX accumulation after LMW HA-DOX co-treatment only in EL4 cells, we analyzed the expression of ABC transporter genes involved in DOX efflux (ABCB1 and ABCG2) only in this cell line. No changes in the expression of ABCG2 mRNA were found during co-treatment with LMW HA and DOX (data not shown). Nevertheless, when EL4 cells were treated with 1 µM DOX, the addition of 20 µg/ml of LMW HA (1.879 ± 0.783) or 100 µg/ml of LMW HA (2.163 ± 0.705) increased ABCB1 mRNA expression respect to DOX alone (Figure 3A). These data are in concordance with the reduction of intracellular accumulation of DOX observed in EL4 in this condition.

EL4 cells were confirmed to have functional pumps since, during the treatment with CsA, DOX accumulation was evidently reduced (Figure 3B). These results indicate that LMW HA may not play a role as a modulator of DOX accumulation and apoptosis in cell lines derived from these solid tumors. However, HA might affect intracellular DOX increase by inducing ABCB1 mRNA expression in hematopoietic malignancies.

### Evaluation of β-catenin and p-Akt expression after LMW HA-DOX co-treatment

Since the modulation of different pathways involved in cell survival and proliferation contributes to carcinogenesis and affects drug response, we analyzed β-catenin and p-Akt expression after the combination of treatments with LMW HA (20 and 100 µg/ml) and DOX (0.5, 1 and 2.5 µM).

**Figure 4:**
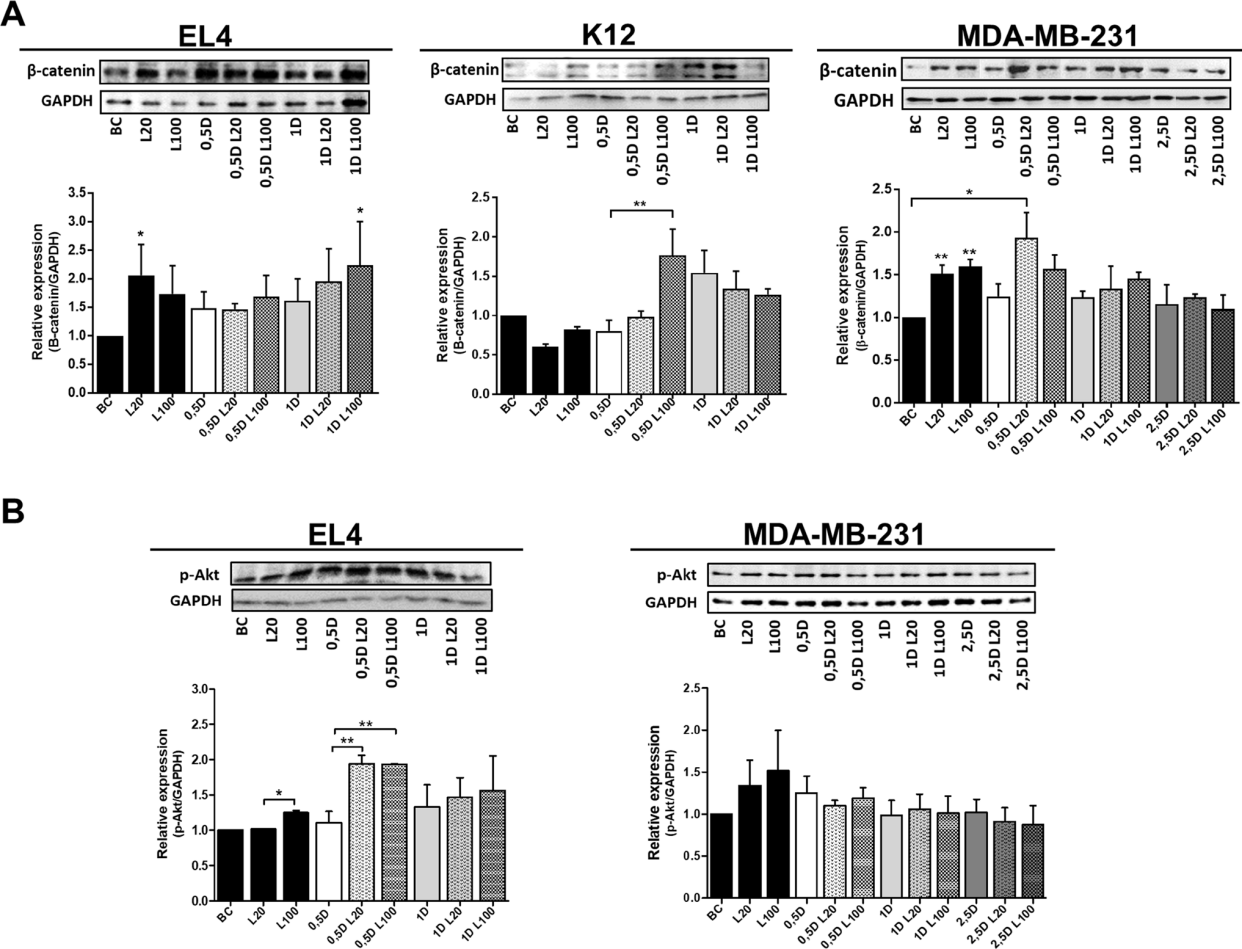
Modulation of β-Catenin and p-Akt expression by DOX and HA co-treatment Protein expression of β-Catenin (**A**) and p-Akt (**B**) was evaluated in EL4, K12 and MDA-MB-231 cells by western blot. Protein extracts were prepared from untreated cells (basal control: BC) or cells treated with HA, DOX, and DOX with HA co-treatment. GAPDH expression was used as loading control. Densitometry analysis of western blot bands was performed, and results are expressed as means ± SD of arbitrary units obtained in three independent experiments. ^*^p < 0.05, ^**^p < 0.01 vs. untreated cells.

In the EL4 cell line treated with different concentrations of LMW HA, β-catenin expression increased, with a significant difference at 20 µg/ml with respect to basal conditions. In turn, DOX treatment increased β-catenin protein levels, standing out at the co-treatment with 1 μM DOX and 100 µg/ml of LMW HA (^*^p < 0.05) (Figure 4A).

Regarding K12 cells, LMW HA treatment did not affect β-catenin expression, but co-treatment with 0.5 µM DOX and 100 µg/ml of LMW HA increased protein expression respect to 0.5 µM DOX (^**^p < 0.01). Treatment with 1 µM DOX also enhanced protein expression, but the addition of LMW HA showed no significant changes (Figure 4A).

Finally, both LMW HA doses significantly increased β-catenin in MDA-MB-231 cells with respect to basal conditions (BC) (^*^p < 0.05) (Figure 4A). Moreover, when cells were treated with 0.5 µM DOX and 20 µg/ml of LMW HA, β-catenin levels were significantly higher (^*^p < 0.05) (Figure 4A). The original nitrocellulose membranes from the three independent experiments for β-Catenin and GAPDH blots are shown in the Supplementary Figure 2. Taken together, these results indicate that LMW HA-DOX co-treatment modulates β-catenin expression in the tumor cell lines studied.

As known, HA stimulation induces PI3K/Akt pathway activation by specific phosphorylation in several tumor cell lines. Thus, and because this activation plays an important role in cancer response to anti-tumoral drugs [26], we decided to evaluate p-Akt expression in the three cell lines.

In EL4 cells, the treatment with 100 µg/ml of LMW HA significantly increased the expression of p-Akt vs. BC (^*^p < 0.05), whereas the treatment with DOX (0.5 µM and 1 µM) induced no differences in p-Akt levels (Figure 4B). When 0.5 µM DOX was combined with 20 or 100 µg/ml of LMW HA, p-Akt levels increased significantly in comparison with 0.5 DOX alone (^**^p < 0.01 and ^**^p < 0.01 respectively). We found similar results with 1 µM DOX in combination with both concentrations of LMW HA. However, no statistically significant differences were found (Figure 4B). These results indicate that LMW HA is capable of reversing the anti-tumoral action of DOX.

In the K12 cell line, we found no detectable levels of p-Akt in the western blot assay under these experimental conditions.

Finally, when we analyzed p-Akt expression in MDA-MB-231 cells, we found an increase in p-Akt expression when cells were treated with 20 and 100 μg/ml of LMW HA (Figure 4B). The original nitrocellulose membranes from the three independent experiments for p-Akt and GAPDH blots are shown in the Supplementary Figure 3. These results suggest that HA treatment favors tumor progression by activating the signaling pathways involved in tumor survival, as was expected. Nevertheless, we observed no differences in p-Akt levels during HA-DOX co-treatment (Figure 4B).

### Modulation of endothelial cell behavior in response to LMW HA-DOX co-treatment

As known, DOX treatment is efficient in inducing tumor cell death. However, in tumor and stromal cells, the tumor microenvironment and its ECM components can impair and modulate these responses, by modulating ECs and thereby angiogenesis [27]. To evaluate whether LMW HA was able to affect the angiogenic response of tumor cells, supernatants from each treatment (LMW HA alone or plus DOX) were collected and stored as described in the materials and methods section. Subsequently, these supernatants were used to perform wound healing and tube formation assays on ECs, as well as to evaluate the expression of soluble pro-angiogenic factors. Controls to discard DOX or HA residual effects on ECs were also performed during each experiment.

**Figure 5:**
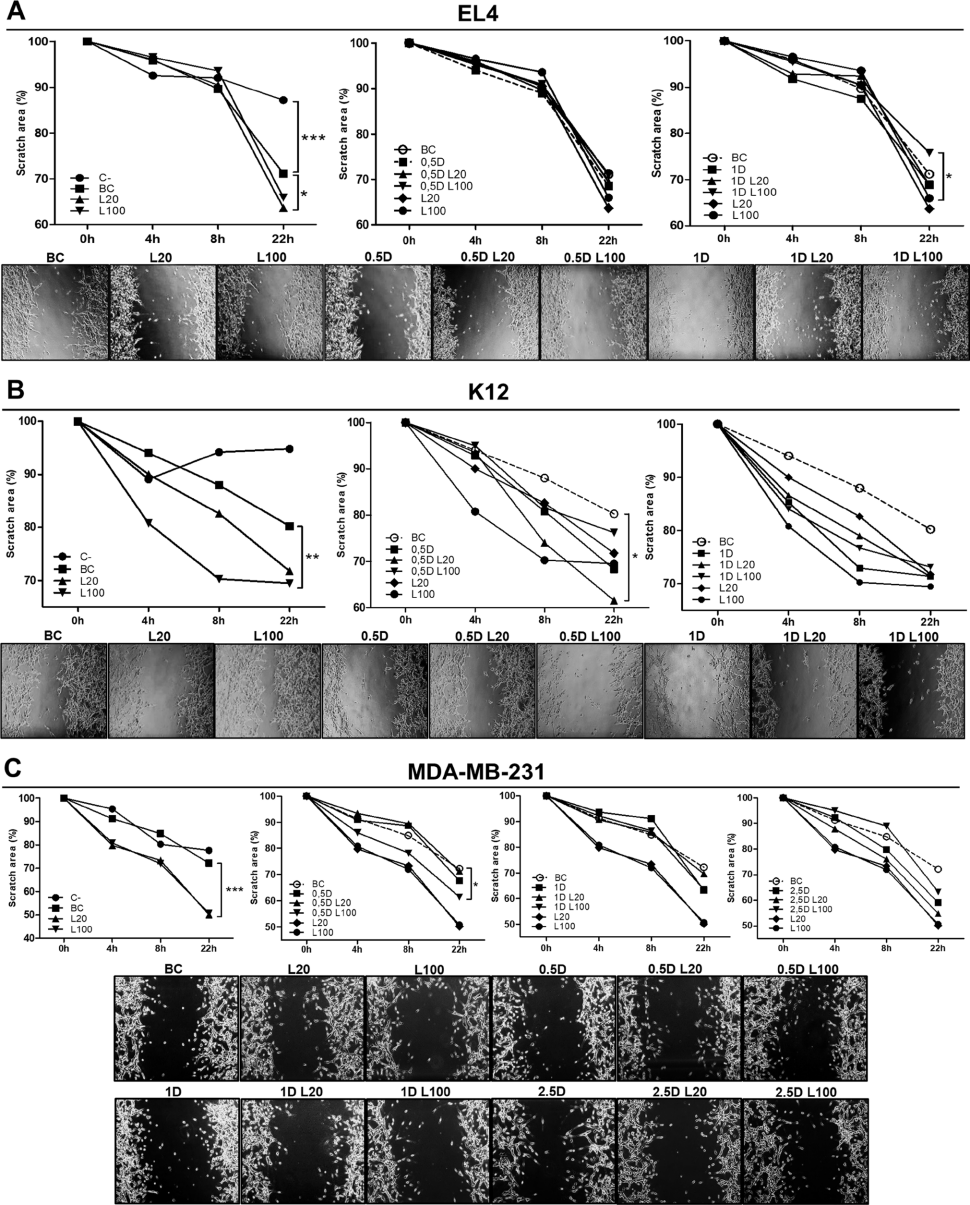
Modulation of endothelial cell migration in response to LMW HA and DOX co-treatment HMEC-1 cells were stimulated with EL4 (**A**), K12 (**B**) or MDA-MB-231 (**C**) supernatants for 24 h. Graphs show the scratch area measured every 4 h. Pictures show the most representative experiment out of three performed. Values are expressed as the arithmetic mean ± SD of three independent experiments. ^*^p < 0.05, ^**^p < 0.01 vs. untreated cells.

Supernatants from EL4 cells treated with 20 µg/ml of LMW HA induced a significant increase in the migration of ECs vs. BC since a reduction of the scratch area was observed (^*^p < 0.05) (Figure 5A). When ECs were stimulated with supernatants from DOX treatments, migration levels were similar to BC, suggesting that DOX itself does not stimulate an angiogenic response in EL4 cells (Figure 5A). However, 100 µg/ml of LWM HA in combination with 1 µM DOX diminished ECs migration compared with HA treatment (^*^p < 0.05) (Figure 5A).

**Figure 6:**
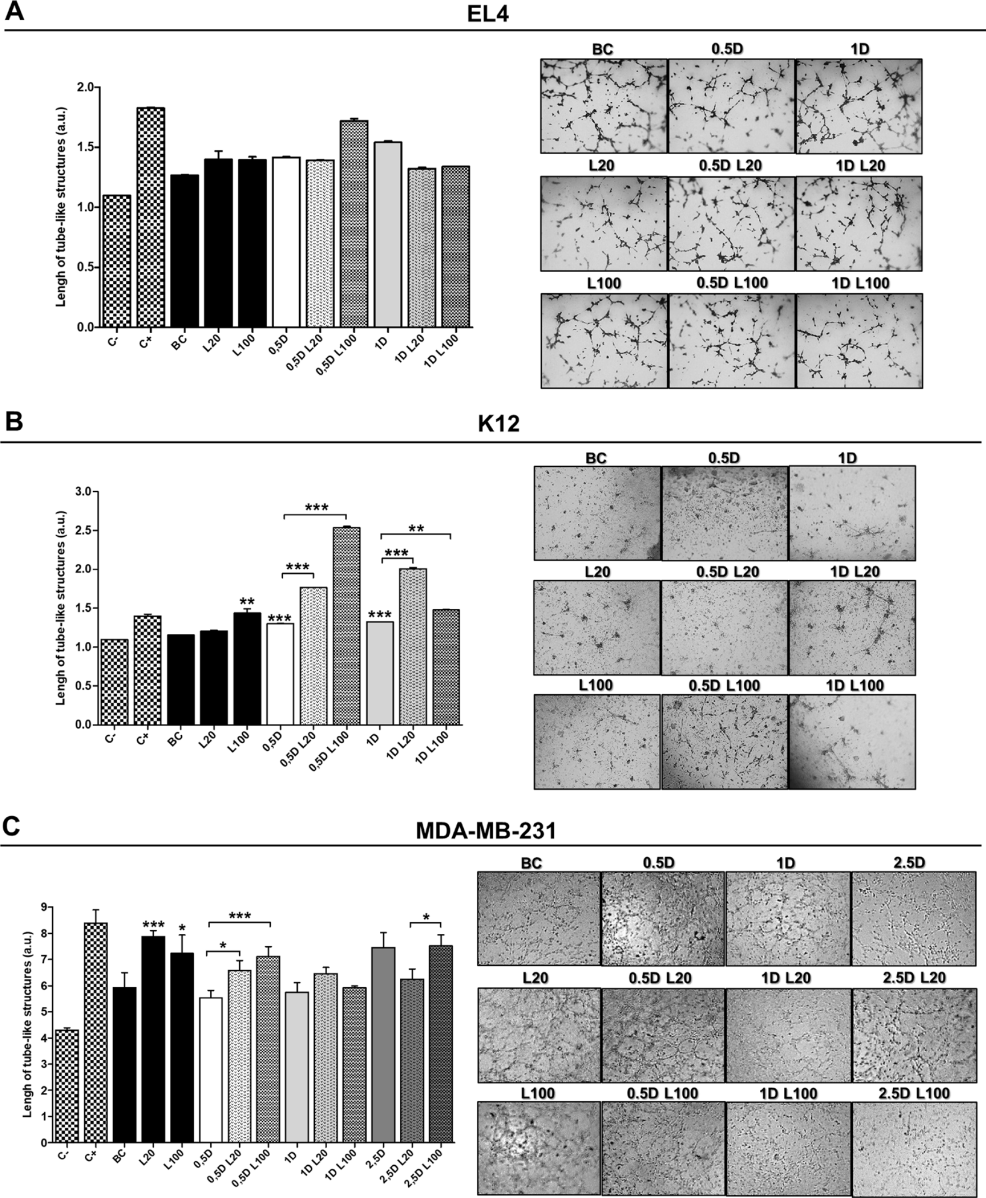
Endothelial cell tube formation assay HMEC-1 cells seeded on a Geltrex™ support were stimulated with EL4 (**A**), K12 (**B**) or MDA-MB-231 (**C**) supernatants. Graphs show the quantification of the arithmetic means ± SD of EC tube formation of three independent experiments. Representative micrographs show the formation of the endothelial network after 6 h of seeding on a Geltrex™ support and stimuli with tumor cells supernatants. C−: DMEM; C+: ECs stimulated with 100 ng/ml of VEGF; BC: supernatant of untreated tumor cells. ^*^p < 0.05 ^***^ *p < 0.001* vs. untreated cells.

To confirm these results, we performed a tube formation assay stimulating ECs in the same conditions as for the wound healing assay. LMW HA treatment (at both 20 µg/ml and 100 µg/ml) appeared to have a pro-angiogenic effect (1.397 ± 0.175 and 1.394 ± 0.06 respectively). Nevertheless, the data were not statistically significant (Figure 6A). Treatment with DOX and its combination with LMW HA did not show an increase in ECs tube formation, indicating that it does not affect the angiogenic behavior of ECs *in vitro* (Figure 6A).

When ECs migration was studied using K12 cell supernatants from LMW HA treatments, as expected, we observed a pro-angiogenic effect. During the wound healing assay, an increase in ECs migration was found when the cells were stimulated with the supernatant of the treatment with 100 µg/ml of LMW HA (^**^p < 0.01) (Figure 5B). In addition, when the tube formation assay was performed, the supernatant of the treatment with 100 µg/ml of LMW HA significantly stimulated ECs tube formation, increasing the number of vessel-like structures respect to BC (1.43 ± 0.05 vs. 1.156 ± 0.03 ^***^p < 0.001) (Figure 6B). DOX treatment itself had a pro-angiogenic action on these tumor cells, since supernatants from the treatments with 0.5 µM and 1 µM DOX induced higher levels of both EC migration (Figure 5B) and tube formation (1.30 ± 0.03 ^***^p < 0.001 and 1.324 ± 0.01 ^**^p < 0.01 respectively vs. BC: 1.156 ± 0.03) (Figure 6B). Besides, we found significant differences when ECs were stimulated by DOX plus LMW HA supernatants in the wound healing assay. Supernatants from the treatment with 5 µM DOX plus 20 µg/ml of LMW HA significantly stimulated EC migration (^*^p < 0.05) (Figure 5B), as well as tube formation (0.5 µM DOX: 1.30 ± 0.03 vs. 0.5 µM DOX + 20 µg/ml LMW HA: 1.76 ± 0.02 and 0.5 µM DOX + 100 µg/ml LMW HA: 2.536 ± 0.01 ^***^p < 0.001) (1 µM DOX: 1.324 ± 0.01 vs. 1 µM DOX+ 20 µg/ml LMW HA: 2.008 ± 0.01 and 1 µM DOX + 100 µg/ml LMW HA: 1.477 ± 0.05 ^**^p < 0.01) (Figure 6B).

Finally, when ECs were treated with HA supernatants from MDA-MB-231 cells, both doses of LMW HA significantly enhanced ECs migration in the wound healing assay (^***^p < 0.001) (Figure 5C). When we used supernatants of these cells treated with DOX, the three doses (0.5, 1 and 2.5 µM) induced an increase in ECs migration compared to BC. Nevertheless, statistically significant differences were found only with 0.5 µM DOX combined with 100 µg/ml LMW HA with respect to BC (^*^p < 0.05) (Figure 5C).

In concordance with the results obtained in the wound healing assay, LMW HA treatment induced a higher formation of tube-like structures in ECs (20 µg/ml of LMW HA: 7.879 ± 0.23 ^***^p < 0.001; 100 µg/ml of LMW HA: 7.226 ± 0.71 ^*^p < 0.05) in comparison with supernatants of untreated MDA-MB-231 cells (BC: 5.928 ± 0.56) (Figure 6C). When ECs were exposed to supernatants from 0.5 DOX plus both LMW HA doses (0.5 µM DOX + 20 µg/ml LMW HA: 6.587 ± 0.37 ^*^p < 0.05 and 0.5 µM DOX + 100 µg/ml LMW HA: 7.108 ± 0.38 ^***^p < 0.001), we found an increase in tube formation in comparison with DOX alone (0.5 µM DOX: 5.535 ± 0.29) (Figure 6C), corresponding with the results obtained in the wound healing assays. Controls to discard residual effects of DOX or HA on ECs were also performed during *in vitro* assays, and results were similar to those for BC (data not shown).

As EL4 cells showed no angiogenic effect compared with K12 and MDA-MB-231 cells in the *in vitro* assays, we decided to evaluate the effect of DOX and HA co-treatment on tumor angiogenesis in an *in vivo* model of murine T-cell lymphoma.

**Figure 7:**
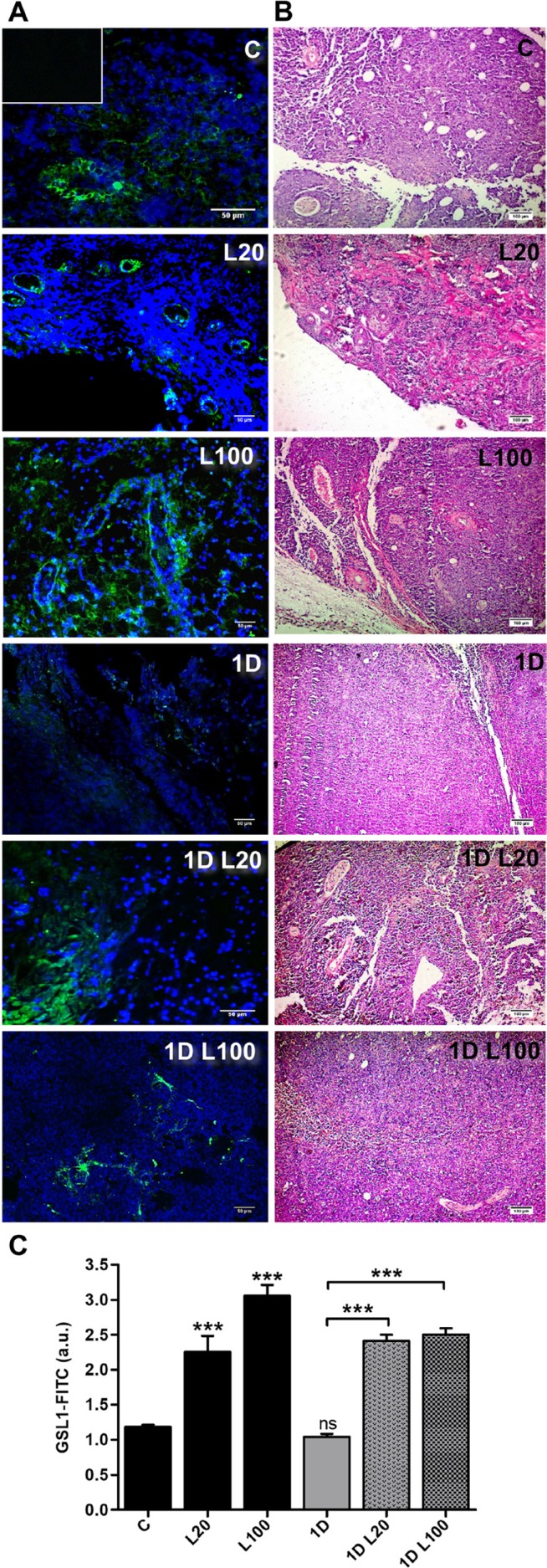
*In vivo* angiogenesis in a model of T-cell lymphoma l Micrographs show the expression of GSL-1-FITC by fluorescence microscope in tumor sections of the EL4 T-cell lymphoma model. C57BL/6 mice were inoculated with EL4 cells and, on day 7, tumors were inoculated s.c. with saline, LMW HA, DOX or DOX plus LMW HA. Tumors were fixed and stained with GSL1-FICT (green, endothelial cells) and DAPI (blue, nuclei) (**A**). Tumors were fixed and stained with hematoxylin/eosin (**B**). Bars represent means of GSL-1-FITC+/field ± SD from ten representative visual fields obtained in three independent experiments (**C**). ^***^p < 0.001 vs. untreated tumors.

Once the s.c. tumor was established, treatments with LMW HA (20 µg/ml and 100 µg/ml), DOX (1 µM), or the combination of both treatments were performed for 48 h. After that time, mice were sacrificed. Subsequently, fluorescent lectin-specific staining was used to determine the presence of ECs and vessels in the tumor tissue. We observed an increase in ECs label (20 µg/ml: 2.254 ± 0.88 and 100 µg/ml:3.057 ± 0.58) and vessel-like structures during the treatment with both doses of LMW HA respect to the tumor tissues from mice that did not receive treatment (1.181 ± 0.05; ^***^p < 0.001) (Figure 7). Contrary to the results obtained *in vitro*, when 1 µM DOX was combined with 20 or 100 µg/ml of LMW HA, an increase in ECs and vessels label was observed (2.412 ± 0.33 and 2.50 ± 0.32 respectively) (Figure 7) comparing to tumor tissues from mice treated with 1 µM DOX alone (1.041 ± 0.17; ^***^p < 0.001). These results could indicate that although angiogenic modulation was not observed *in vitro*, LMW HA disturbs DOX action, up-regulating the angiogenesis process during this therapy.

### Expression of pro-angiogenic factors after LMW HA-DOX co-treatment

**Figure 8:**
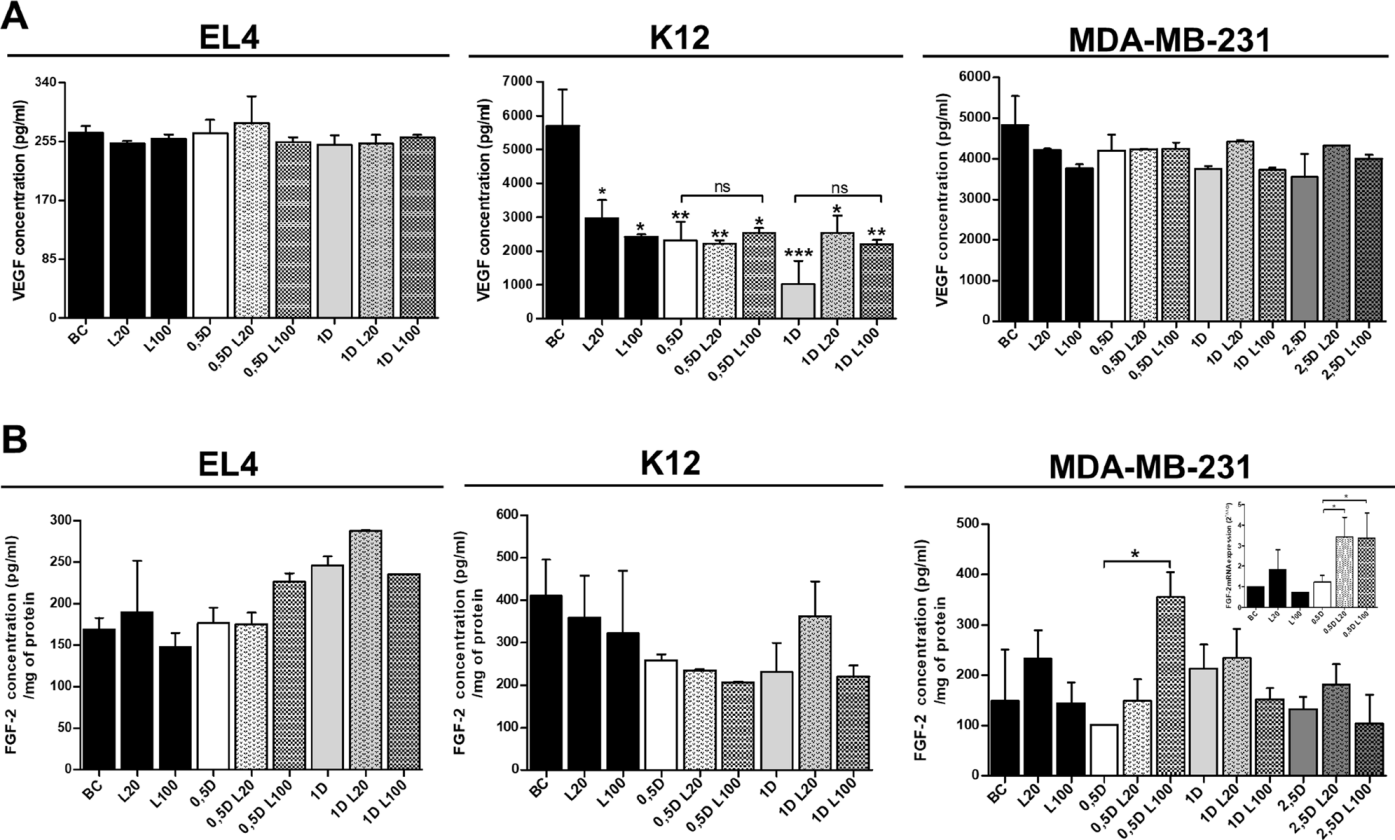
Analysis of pro-angiogenic factors expression after LMW HA and DOX co-treatment A VEGF (**A**) and FGF-2 (**B**) concentration levels (pg/ml) were detected in EL4, K12 and MDA-MB-231 supernatants by ELISA. Results are expressed as means ± SD obtained in three independent experiments. ^*^p < 0.05, ^**^p < 0.01 vs. untreated cells.

Since VEGF is one of the most important factors involved in angiogenesis [28–30], we next analyzed whether VEGF could be one of the soluble factors involved in the modulation of angiogenesis under HA and DOX co-treatment. To this end, we evaluated VEGF secretion in tumor cell supernatants after DOX and LMW HA treatments. When VEGF concentration was measured in EL4 cells (Figure 8A), no significant differences were observed between treatments. In contrast, and unexpectedly, when VEGF expression was evaluated in K12 cells, a significant reduction of VEGF levels was observed in all treatments, compared to BC (Figure 8A). When VEGF levels were measured in MDA-MB-231 supernatants, no difference in protein concentration was observed (Figure 8A).

Considering that no differences in VEGF expression were detected in supernatants of tumor cells, other factors could be involved in tumor angiogenesis and be related to aggressive phenotypes of different types of cancer cells [31–33]. Since, FGF-2 is a potent cell survival factor involved in tumor angiogenesis [33–35] we decided to evaluate FGF-2 expression in EL4, K12, and MDA-MB-231 cells. First, we analyzed FGF-2 protein expression by using supernatants from the three cell lines after performing all treatments with LMW HA and DOX. However, no detectable levels were found by ELISA. Because FGF-2 is not frequently detected into the medium of cultured cells because remains associated with cell-surface heparan sulfate proteoglycans (HSPGs) upon secretion [36–38], we performed the ELISA with total cellular protein extracts from each cell line.

When we evaluated the biosynthesis of FGF-2 in EL4 cells, treatment with 20 µg/ml of LMW HA increased FGF-2 expression levels, although no significant differences were found. When 0.5 µM DOX was combined with 100 µg/ml of LMW HA, an increase in FGF-2 levels was observed compared with 0.5 µM DOX alone (*p = 0.055*). In addition, the treatment with 1 µM DOX and 20 µg/ml of LMW HA also showed higher levels of FGF-2 than the treatment with 1 µM DOX alone (not significant) (Figure 8B).

Conversely, no changes in FGF-2 expression were observed when K12 cells were treated with LMW HA (Figure 8B). When 1 µM DOX was combined with 20 µg/ml of LMW HA, FGF-2 levels increased with respect to treatment with 1 µM DOX alone (Figure 8B).

Since the highest pro-angiogenic effect was observed in MDA-MB-231 cells, we decided to evaluate both mRNA and protein levels of FGF-2 after LMW HA and DOX treatments. The treatment with 20 µg/ml of LMW HA induced a significant increase in FGF-2 mRNA levels (Figure 8B). Furthermore, the LMW HA plus DOX treatment induced a higher expression of FGF-2 than DOX treatment alone (Figure 8B). When we studied the biosynthesis of FGF-2 by ELISA, the treatment with 20 µg/ml of LMW HA increasedFGF-2 levels. In agreement with our previous results, DOX treatment *per se* showed no pro-angiogenic action. However, when 0.5 µM DOX was combined with 100 µg/ml of LMW HA, FGF-2 expression increased significantly in comparison with 0.5 µM DOX alone (^*^p < 0.05) (Figure 8B). These results can explain the pro-angiogenic effect on ECs detected in all *in vitro* assays using MDA-MB-231 cells (Figures 5 and 6).

Our results demonstrate for the first time that HA is a potential modulator of the angiogenic response in combination with DOX treatment. The effect of adding HA to DOX treatment is related to the modulation of the expression of pro-angiogenic factors, which finally increase EC migration. Besides, we also showed that a chemotherapeutic drug such as DOX could affect tumor angiogenesis by modulating the expression of the angiogenic factor FGF-2.

## DISCUSSION

In cancer, one of the main causes of recurrence and mortality is the development of drug resistance. The tumor microenvironment is formed by different populations of cells (cancer and stromal cells) and ECM components, whose interactions influence the sensitivity to apoptosis and affect drug response, potentially inducing the appearance of resistance to chemotherapy [39].

In the present study, our objective was to elucidate whether the interaction of HA with tumor cells from different histological origin affects the response to the antineoplastic drug DOX and in turn the angiogenesis process, determining a general or differential action of HA within these tumors. To this end, we used cell lines derived from hematologic cells (EL4 cells), epithelial breast adenocarcinoma (MDA-MB-231 cells) and mesenchymal tumors (osteosarcoma K12 cells). We first analyzed CD44 expression and its ability to bind HA in all tumor cell lines to discard a different behavior as a consequence of the loss of CD44 expression. Although there are other HA receptors, we focused only on the interaction with CD44 because it is the main receptor involved in tumor processes [40, 41]. In fact, it has been reported that the HA-CD44 interaction is associated with the high tumorigenesis of MDA-MB-231 cells [22]. Several reports have indicated that CD44 expression contributes to aggressive progression in osteosarcoma [42] as well as in hematologic malignancies [43, 44]. Here, the three tumor cell lines evaluated showed high levels of CD44 expression, detecting two different populations (*with low and high expression*). Besides, the cells bound LMW HA efficiently, and different cell populations were observed in each tumor cell line when HA binding was analyzed, clearly showing either two or even three subpopulations that bound HA with different ability. Although the exact biological significance of this finding is not fully clear, these differences could partly explain the differential behavior found during HA treatments in the tumor cell lines evaluated. We will thus continue investigating these cell subpopulations to better understand the connection with the different responses observed during drug treatments in patients, since this might be associated with the therapy resistance and/or relapse of cancer disease.

It is well known that drug resistance can arise through several mechanisms, including an increase in drug efflux via the modulation of cell membrane multidrug resistance transporters [6, 45, 46]. It has been demonstrated that ECM components, such as HA, modulate drug response, affecting the expression and function of different drug efflux transporters [47, 48].

In the present study, tumor cells were exposed to DOX because this compound is widely used as an antineoplastic drug for a broad spectrum of tumors [49]. To discard drug action over cell viability during the treatments, we used different DOX doses (0.5 µM, 1 µM and 2.5 µM), at which apoptosis changes were minimal. HA treatment induced a decrease in DOX intracellular levels only in T-cell lymphoma cells, but this decrease did not impact cell survival as apoptosis levels were similar during HA-DOX co-treatment respect to the control or DOX treatment alone. As expected, these results are a consequence of the modulation of the expression of ABC drug transporters involved in DOX efflux, as we observed increased ABCB1 mRNA expression. In contrast, our results indicated that LMW HA could not play a role as a modulator of DOX accumulation in cells derived from solid tumors, although these cells have active ABC drug transporters. When we analyzed the presence of functional pumps in all cell lines by using CsA, an inhibitor of ABC transporters which blocks the function of ABCB1 and ABCC1 pumps, we observed a reduction of DOX accumulation when the cells were co-treated with CsA + DOX (data not shown). The results suggest that ECM components from hematologic tumors, specifically HA, could impact on DOX response, generating subpopulations of resistant cells. HA might be involved in the conversion of cancer cells into cancer-initiating cells or cancer stem cells [10, 24], characterized by high expression of ABC transporters and other cancer stem cell markers such as CD44 [7].

On the other hand, it has been recently demonstrated that mammary tumors show an increased chemoresistance to DOX, which is due not only to a reduction of drug internalization, but also to specific interactions with the tumor microenvironment. Specifically, it has been determined that the “*tissue phenotype*” or the ECM generated around the tumor is responsible for inducing survival mechanisms that evade drug response [50].

It is well known that the modulation of cell survival and proliferation pathways contributes to carcinogenesis and drug resistance [11, 39, 51]. Considering these facts, we studied the effect of DOX and HA treatment on the Wnt/β-catenin and PI3K/Akt pathways. Wnt/β-catenin signaling favors pathological angiogenesis by regulating the expression of VEGF [52] and interleukin-8 [53]. Moreover, in a gastric cancer model, it has been reported that, upon DOX chemotherapy, the Wnt pathway is activated, promoting tumor invasion and metastasis [54]. In the present study, our results indicated that DOX treatment in combination with LMW HA enhanced β-catenin expression by additive effect, as observed in EL4 and MDA-MB-231 cells. HA and DOX alone had the ability to modulate the expression of β-catenin, whereas the co-treatment with DOX and HA enhanced this modulation. However, only the HA-DOX co-treatment induced the modulation of β-catenin expression in K12 cells. As mentioned above, this could be the consequence of the differential CD44 expression and HA binding ability observed in the different cell lines studied. Nevertheless, these findings showed that HA could be involved in DOX response, impairing the effect of the drug and favoring tumor progression mediated by Wnt/β-catenin signaling.

We also explored the activation of Akt protein since an aberrant activation of this pathway is involved in cancer progression and resistance to chemotherapy [55–57]. As expected, LMW HA treatment increased p-Akt expression, showing that HA could be favoring the activation of this pathway in EL4 and MDA-MB-231 cells. However, when cells were treated with DOX, these hematologic and epithelial tumor cells showed a different behavior. LMW HA was able to reverse the anti-tumoral action of DOX in EL4 cells, besides the DOX effect was independent of the presence of HA. This result allowed us to rule out an HA-DOX tumor modulation by this pathway in MDA-MB-231 cells. p-Akt expression was not detected in K12 cells in our experimental conditions, although we used an antibody that detected all p-Akt isoforms. Thus, we cannot elucidate the role of p-Akt in the osteosarcoma cell line in association with HA-DOX tumor modulation.

Some studies have shown that cancer cells protect ECs from apoptosis after radiation through secretion of VEGF and subsequent activation of pro-survival pathways [58]. Taking these data into account, we explored an unwanted effect observed during chemotherapeutic treatment: modulation of angiogenesis. In the tumor microenvironment, HA regulates angiogenesis, and specifically, LMW molecules have a pro-angiogenic action [59, 60]. We hypothesized that LMW HA from tumor stroma could also affect the anti-angiogenic action of DOX observed in ECs [61, 62]. Published data have shown that DOX induces a differential resistance in tumor-associated ECs [27, 63]; nevertheless, the potential mechanisms involved have not been extensively explored. As expected, LMW HA enhanced the angiogenic action of the supernatant derived from tumor cells and, surprisingly, DOX showed a similar effect. When DOX treatment was combined with HA, both EC migration and vessel formation *in vitro* were enhanced. This suggests that HA might favor tumor progression and alter a suitable response to DOX, enhancing angiogenesis in epithelial and mesenchymal derived tumor. Although opposite results were observed for hematologic tumor cells *in vitro*, detection of vessel formation in our *in vivo* model revealed that DOX might also affect angiogenesis in the presence of HA in lymphoma tumors. This important finding confirms that the tumor ECM could reduce the success of chemotherapy in different types of tumor.

Since VEGF is involved in tumor angiogenesis, we first explored mechanisms by which HA modulates VEGF levels [13]. However, we did not find a significant increase in VEGF secretion; in fact, a decrease in this factor was observed in K12 cells. These results could be explained by two findings. First, VEGF165 exists in different isoforms: angiogenic VEGF165a and anti-angiogenic VEGF165b, and this proportion could be modulated. For example, selective binding of VEGF165a by C-6 OH sulfated HA affects VEGF action, enhancing the anti-angiogenic effect of VEGF165b [64]. Thus, we can hypothesize that the reduction of VEGF in K12 supernatants could be related to its anti-angiogenic VEGF function. Second, there are other angiogenic factors released by tumor cells, which can modulate EC behavior independently of VEGF biosynthesis. Thus, in the absence of modulation of VEGF expression in EL4 and MDA-MB-231 cells, the observed effect could be due to some other potent angiogenic molecule. These results could explain that therapies focused on the down-regulation of VEGF expression or associated cellular signals fail as a consequence of changes in the tumor microenvironment and bio-availability of other important angiogenic factors [31, 33, 65].

Actually, FGF-1 and FGF-2 have been reported to be up-regulated in tumors that relapsed from the treatment with an anti-VEGFR antibody [66]. In the present study, DOX treatment caused an up-regulation of FGF-2 biosynthesis, which might be associated with the pro-angiogenic effect observed in combination with HA in all the cell lines evaluated. It is well known that the mainly fraction of the FGF-2 produced by the tumor cells is secreted and remain bind to the heparan sulfate proteoglycans in the cytoplasmic membrane. Therefore, it could be detected in the cellular lysis extracts instead of in the supernatants. This fraction of FGF-2 is the mainly isoform detected by ELISA [37]. Besides, we performed the ELISA with the supernatants from the tumor cells treated with HA + DOX to rule out that the addition of HA had affected the interaction between the heparan sulfate proteoglycans and FGF-2, and releasing it into the cell medium. We found detectable levels of FGF-2 only in the ELISA performed from the cell extracts, therefore our results could be related to FGF-2 isoforms retain in the cytoplasmic membrane in interaction with ECM components. In addition to the angiogenic effect of FGF-2, certain FGF-2 isoforms that are not secreted from the cell and are transported to the nucleus where they regulate cell growth or behavior [67, 68].

Our results demonstrate for the first time that LMW HA is a potential modulator of the angiogenic responses by interaction with the chemotherapeutic drug DOX. HA in combination with DOX treatment increased EC migration and formation of vessel-like structures. These results could indicate a different and new mechanism by which HA from the tumor ECM could modulate DOX response in tumor cells, raising an unwanted effect of this drug: promotion of angiogenesis.

## MATERIALS AND METHODS

### Reagents

Recombinant LMW HA (1–3 × 10^5^ CPN Czech Republic) was kindly supplied by Farmatrade (Argentina). High glucose Dulbecco’s modified Eagle’s medium (DMEM) and DMEM F12 were purchased from MICROVET Laboratories (Argentina). TriReagent was from Molecular Research Center, Inc (USA). Doxorubicin (DOX) was kindly provided by Filaxis Pharmaceuticals S.A (Argentina). Anti-β-catenin antibody was purchased from Millipore (USA). Specific antibody against glyceraldehyde-3-phosphate dehydrogenase (GAPDH) antibody was from NeoBioLab (USA). Anti-phosphorylated Akt (Ser473, Ser472 and Ser474) antibody was purchased from R&D System (USA) and anti-rabbit secondary horseradish peroxidase (HRP) antibody was from Santa Cruz Biotechnology (USA). CD44-APC antibody was from BD BioSciences (USA) and HA-FITC from Calbiochem (USA). Annexin V-FITC apoptosis detection kit was from ImmunoTools (Germany). Cyclosporine A was kindly provided by Novartis Pharmaceuticals Corporation (Argentina).

### Cell lines

The murine T-cell lymphoma cell line EL4 (TIB-39™) and MDA-MB-231 human breast cancer cell line (HTB-26™) were purchased from ATCC^®^ (USA). K12 osteosarcoma cells were provided by Dr. Eugenie Kleinerman (University of Texas M.D. Anderson Cancer Center, Houston, TX, USA). The HMEC-1 cell line was gently provided by Dr. Candal (Centers for Disease Control, Atlanta, USA). The MDA-MB-231 human cell line was authenticated by Northgene Ltd. Company (UK), using highly sensitive DNA testing for Short Tandem Repeats The murine cell lines (EL4 and K12) were also analyzed to identify each cell line and rule out cross contamination with human cell lines by the Quality Control Department of the National Institute of Human Viral Diseases (INEVH), Argentina. EL4, K12 and HMEC-1 cells were maintained in high glucose DMEM supplemented with 10% heat-inactivated fetal bovine serum (FBS), 2 mM L-glutamine, 100 U/ml streptomycin, and 100 mg/ml penicillin, and incubated at 37° C in a 5% CO2 atmosphere. Similarly, MDA-MB-231 cells were cultured with DMEM F12 supplemented with 10% FBS, 2 mM L-glutamine, 100 U/ml streptomycin and 100 mg/ml penicillin. During all cell cultures, periodic checkups of cell morphology as well as strict control of cell line passages (5–10th passage) and cell line growth rate were performed. In addition, all cell lines were analyzed to discard the presence of mycoplasma contamination by PCR assay.

### CD44 receptor expression and HA binding ability

To determine CD44 expression levels and HA binding ability, 5 × 10^5^ cells were incubated with 20 μg/ml of HA-FITC or anti-CD44-APC monoclonal antibody in 100 μl of 1% bovine serum albumin (BSA) in phosphate buffered saline (PBS) for 1 h at 4° C and washed twice with 1% BSA-PBS. The analysis was performed on FACS Canto II flow cytometer (BD Biosciences). Data were analyzed using FlowJo software (LLC).

### HA and DOX treatments

HA diluted in ultra-pure water and used up to a concentration of 6 mg/ml. HA concentrations used in the experiments were 20 μg/ml and 100 μg/ml. DOX was diluted in sterile saline solution to 40 μM, and used in doses of 0.5 μM, 1 μM and 2.5 μM. Cell lines were cultured in 12-well plates for 24 h before treatment. The next day, all supernatants were removed and fresh culture medium without FBS was added to each well. After that, LMW HA was added to the medium for 24 h, and after 12 h of this treatment, DOX was added for the remaining 12 h of HA treatment. Subsequently, supernatants were collected and conserved at −80° C until their use.

### DOX accumulation assay

Since DOX has an emission spectrum detectable by flow cytometry between 550–600 λ, DOX efflux was analyzed measuring intracellular drug accumulation as previously described [11]. Cells (5 × 10^5^) were treated as mentioned above and DOX fluorescence was collected through a 564–606 nm band-pass filter. To study the presence of functional drug efflux pumps in all cell lines, we also studied DOX accumulation in the presence or absence of 100 µM of the inhibitor CsA. Samples were analyzed using FACS Canto II Flow cytometer and data were evaluated using FlowJo software.

### Apoptosis detection assay

To evaluate apoptosis levels induced by DOX treatments, the Annexin V-FITC apoptosis detection kit (ImmunoTools) was used, following the manufacturer´s protocol. Samples were analyzed using FACS Canto II Flow cytometer and data were evaluated using FlowJo software.

### RT-qPCR

After each treatment with DOX and HA in EL4 and MDA-MB-231 cells, total RNA was extracted by Tri Reagent (Sigma-Aldrich Co). RNA integrity and quantification were assessed by spectrophotometry, measuring OD260 in a Picodrop^®^ instrument. Two micrograms of RNA were reverse-transcribed with 200 U of RT M-MLV Reverse Transcriptase (Promega) and 2.5 pmol/µl of Oligo (dT) primers (GenBiotech). cDNAs were then subjected to real-time quantitative PCR (RT-qPCR) using FastStart SYBR Green Master (Roche) and 200 nM of each specific primer (Invitrogen): murine ABCB1 forward 5′-CTG GTT TGA TGT GCA TGA CG-3′ and reverse 5′-GAA CAT TCC GAT TTT GTC ACC-3′; murine ABCG2 forward 5′-TCG CAG AAG GAG ATG TGT TG-3′ and reverse 5′-TGG GTC CCA GAA TAG CAT TAA G-3′; human FGF-2 forward 5′-CCTGGCTATGAAGGAAGATGG 3′ and reverse 5′ TCGTTTCAGTGCCACATACC 3′ and human EGF forward 5′ TGA TAA GCG GCT GTT TTG G-3′ and reverse 5′-CAC CAA AAA GGG ACA TTG C-3′. PCR conditions were 90 seconds at 94° C and then 40 cycles of 30 seconds at 94° C, 30 seconds at 60° C and 30 seconds at 72° C. Values were normalized to levels of murine and human glyceraldehyde-3-phosphate dehydrogenase (GAPDH; used as housekeeping) transcript (forward 5′-GGG GCT GCC CAG AAC ATC AT-3′ and reverse 5′-GCC TGC TTC ACC ACC TTC TTG-3′). A non-template control was run in every assay and all determinations were performed in duplicate in three separate experiments.

### Protein extracts and western blot analysis

To analyze β-catenin and p-Akt expression, tumor cell lines were treated with HA and DOX (1 × 10^6^) as described above and then lysed with lysis buffer for 30 minutes at 4° C [69]. After centrifugation of cells, supernatants were preserved, and protein concentration was measured using Bradford protein assay. Protein extracts were stored at −80° C until use. Equal amounts of protein were resolved by 0.1% SDS-10% polyacrylamide gel denaturing electrophoresis (SDS-PAGE) and transferred onto a nitrocellulose membrane. The membranes were incubated with a specific anti-β-catenin or p-Akt antibody and GAPDH overnight at 4° C, and then incubated with horseradish peroxidase-labeled secondary antibody for 1.5 h at room temperature. Finally, HRP chemiluminescence reaction was detected using a stable peroxide solution and an enhanced luminol solution. Images were obtained with an ImageQuant 4000 mini bioluminescent image analyzer (GE HealthCare LifeSciences) and analyzed using ImageJ 1.50 b software package (National Institutes of Health, USA).

### Endothelial cell wound healing assay

HMEC-1 micro-endothelial cells were grown to confluence on 24-well plates. Then, 18 hours before starting the assay, HMEC-1 cells were subjected to FBS starvation to avoid proliferation effects. Consistently shaped wounds were made using a sterile 100-µl pipette tip across each well, creating a cell-free area line [70]. At that point, cells were exposed to the supernatants of EL4, K12 or MDA-MB-231 cells (previously treated with LMW HA, DOX, or LMW HA + DOX) diluted at 1:1 ratio with DMEM. For negative control, cells were exposed to DMEM without FBS. Controls to discard residual effects of DOX or HA on ECs were also performed. Three images were captured in the same coordinates point at 0, 4, 8 and 22 h after performing the wound. The gap size of the wound was measured and analyzed using ImageJ 1.50b software package.

### Endothelial cell tube formation assay

A tube formation assay was performed using Geltrex™ LDEV-Free reduced growth factor basement membrane matrix (GibcoTM Life Technologies) [71]. To this end, 40 μl of Geltrex™/well was seeded in a 96-well plate and allowed to polymerize for 30 minutes at 37° C. HMEC-1 cells (2 × 10^4^), FBS-starved 18 h before, were loaded into GELTREX and exposed to the supernatants of EL4, K12 and MDA-MB-231 cells from the different treatments. For positive control, 100 ng/ml of recombinant human VEGF was used to stimulate tube formation of HMEC-1 cells, whereas for negative control, only DMEM medium was used. After 6 h of incubation at 37° C, cells were stained using eosin solution. Quantification was performed by analyzing the cell-free area from five images per well with the ImageJ 1.50 b software package.

### VEGF and FGF-2 ELISAs

Human and mouse VEGF secretion levels were determined by the DuoSet ELISA Kit (R&D System, USA) from free-cell culture supernatants after treating cell lines with DOX and HA. FGF-2 expression levels were determined by the DuoSet ELISA Kit (R&D System, USA) from protein extracts of all cell lines treated with DOX and HA. The assays were carried out according to the instructions provided by the manufacturer.

### *In vivo* experiments

EL4 cells were s.c. injected, at a dose of 1 × 10^6^ cells/animal, into the right flank of four-to six-week-old C57BL/6 mice. Tumors were allowed to reach approximately 85 mm^3^ in size before DOX and HA treatments were started. Animals were distributed in different groups, and then s.c. treated on day 7 after tumor inoculation with saline, 20 μg/ml or 100 μg/ml of LMW HA, 1 μM of DOX, 1 μM of DOX plus 20 μg/ml of LMW HA or 1 μM of DOX plus 100 μg/ml of LMW HA. After 2 days, on day 9, mice were sacrificed, and tumors were removed, fixed in 4% formalin and embedded in paraffin. Before staining, 3-μm sections were deparaffinized and dehydrated. Slides were rinsed with PBS and dyed with DAPI 0.3 µg/ml plus fluorescein-labeled Griffonia (Bandeiraea) Simplicifolia Lectin I 20 µg/ml (GSL I, Vector Laboratories # FL-1101), which binds specifically to ECs in mouse tissues [72]. Furthermore, sections for histological analysis were routinely stained with hematoxylin/eosin. The sections were rinsed with PBS and then mounted on microscope slides. Micrographs of the stained sections were taken with a Nikon Eclipse E800 fluorescence microscope. Images were analyzed with the ImageJ 1.50b software package.

### Statistical analysis

For statistical analysis, 95% confidence intervals (CI) were determined by calculating arithmetic mean values and variance (standard deviation, SD) of three independent experiments. To evaluate whether differences between the values obtained were significant, the T Student’s test (*T*-test, Mann–Whitney) was used in the case of comparisons between two groups. Analysis of variance (ANOVA, Tukey Test) was also used to evaluate the differences between values of more than two experimental groups. The software Prism (GraphPad, San Diego, CA, USA) was used, considering a *p* value < 0.05 as statistically significant.

## SUPPLEMENTARY MATERIALS FIGURES



## Abbreviations

DOX: Doxorubicin
ECs: Endothelial cells
FGF: Fibroblast growth factor
HA: Hyaluronan
HRP: Horseradish peroxidase
LMW: Low molecular weight
VEGF: Vascular endothelial growth factor
FGF-2: Fibroblast growth factor 2
