# Supplementary material for: Co-treatment of tumor cells with hyaluronan plus doxorubicin affects endothelial cell behavior independently of VEGF expression
Source: Oncotarget. 2018 Nov 27;9(93):36585–602. doi: 10.18632/oncotarget.26379 (PMC6290962; doi:10.18632/oncotarget.26379)
Supplement: Supplementary file 1 [file oncotarget-09-36585-s001.pdf]

Co-treatment of tumor cells with hyaluronan plus doxorubicin affects endothelial cell behavior independently of VEGF expression

SUPPLEMENTARY MATERIALS

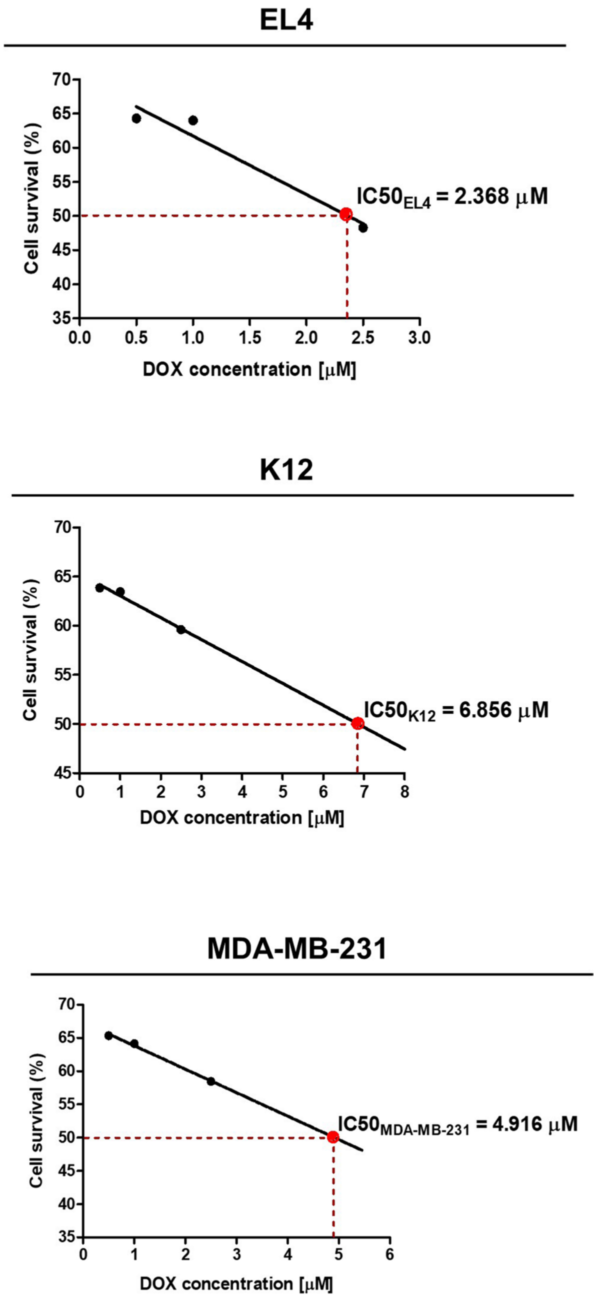

Supplementary Figure 1: Determination of IC50 values for DOX in tumor cell lines. EL4, K12, MDA-MB-231 cells were treated in triplicate wells for 12 hours with varying concentrations of DOX. Following treatment, cell survival was determined by Annexin V assays. Each data point represents the average of values obtained in 3 independent experiments. IC50 values (μM) were determined using the software Prism (GraphPad, San Diego, CA, USA).

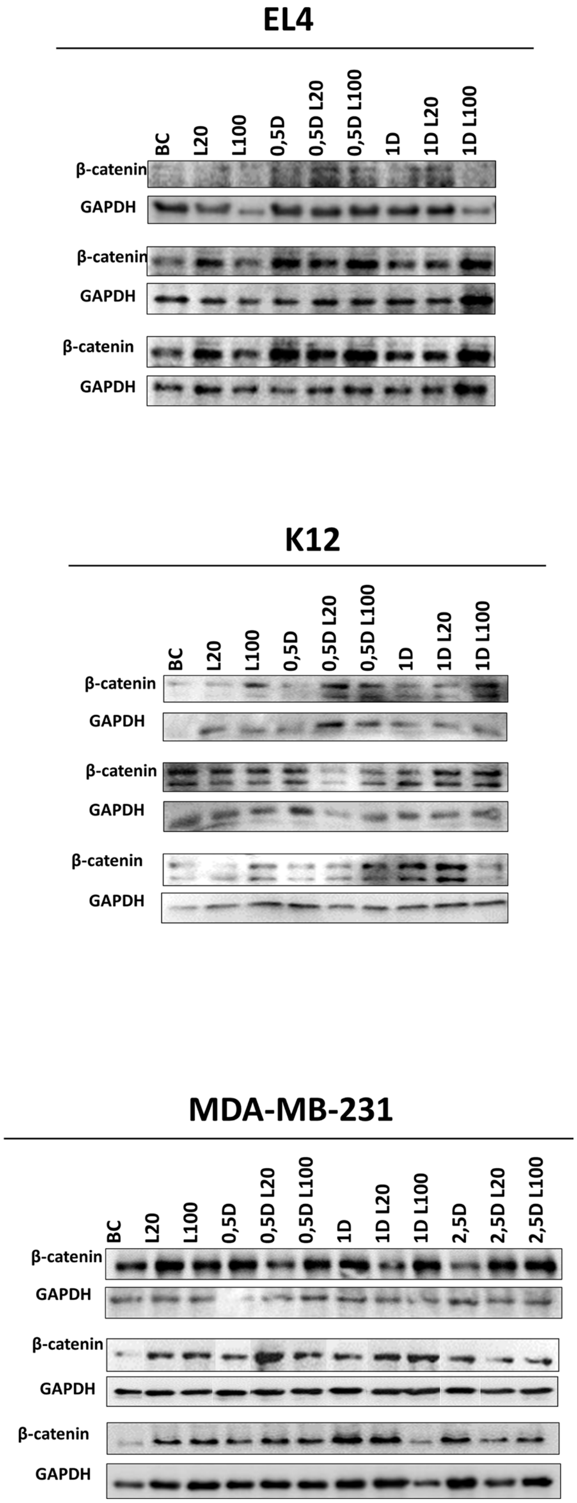

Supplementary Figure 2: Original nitrocellulose membranes from β-Catenin western blot. Protein expression of β-Catenin evaluated in EL4, K12 and MDA-MB-231 cells in 3 independent western blot membranes. The proteins levels were quantified as described in Material and Methods.

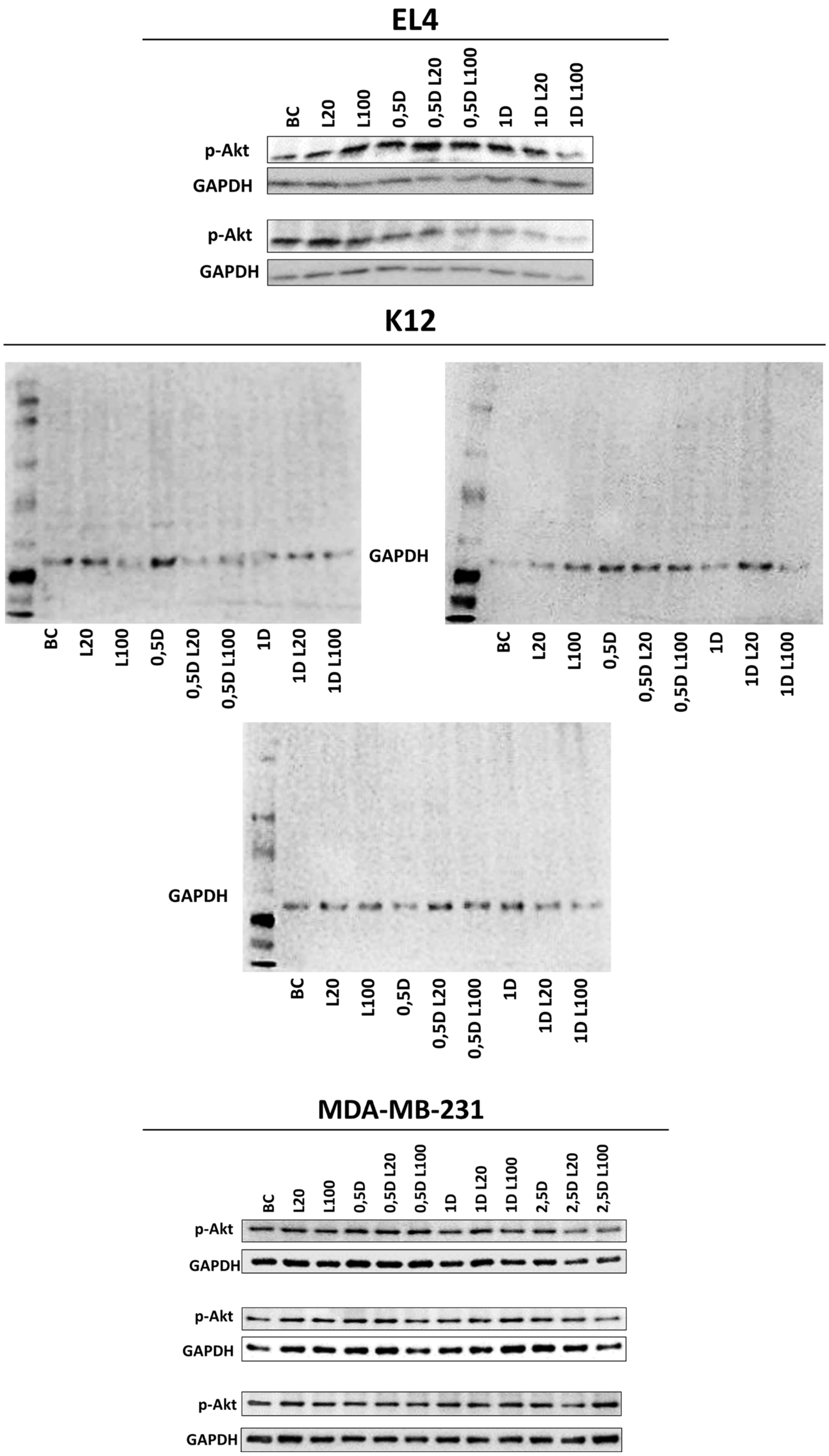

Supplementary Figure 3: Original nitrocellulose membranes from p-Akt western blot. Protein expression of p-Akt evaluated in EL4, K12 and MDA-MB-231 cells in 3 independent western blot membranes. The proteins levels were quantified as described in Material and Methods.
